# Supplementary material for: CRISPR-induced double-strand breaks trigger recombination between homologous chromosome arms
Source: Life Sci Alliance. 2019 Jun 13;2(3):e201800267. doi: 10.26508/lsa.201800267 (PMC6587125; doi:10.26508/lsa.201800267)
Supplement: Supplementary file 1 [file LSA-2018-00267_TableS1.docx]

**Table S1:** Shown are the readable sequences (n=84) for recombination on the X chromosome (n_total_=104); molecular analysis of the target site. Deleted bp shown as: –

Inserted/exchanged bp shown as: N

Bold, grey sequence numbers show unrearranged (un-CRISPRed) CIGAR reporters.

To facilitate the detection of recombination, the first four nucleotides of the shifter of CIGAR^eGFP^ (CGGC) and CIGAR^mCherry^ (CCCC) are highlighted. The **PAM** site is shown in bold letters. In addition, we highlight the PCR setup used to amplify the shifter sequences of CIGAR^eGFP^ (green) or CIGAR^mCherry^(red). The fwd primer anneals within the ubiquitin promoter (identical between both CIGAR variants), and the reverse primers primer are either specific for eGFP or mCherry, respectively.

The used primers for CIGAR^eGFP^: CIGAR-fwd: CAACAAAGTTGGCGTCGATA and CIGAR^eGFP^ -rev: GAACTTCAGGGTCAGCTTGC.

The used primers for CIGAR^mCherry:^ CIGAR-fwd: CAACAAAGTTGGCGTCGATA and CIGAR^mCherry^ -rev: AAGCGCATGAACTCCTTGATG.

Orig CIGAR^eGFP^ GTGCGGCGACAGCAGAACGTAGC**GGG**ACGATAGGCTGCAG

|||| ||| | || || | |**|||**||||||||||||||

Orig CIGAR^mCherry^ GTGCCCCGAGACAAGCACCTGAC**GGG**ACGATAGGCTGCAG

1 Recombination GTGCCCCGAGACAAGCACCTGAC**GGG**ACGATAGGCTGCAG PCR

2 CIGAR^eGFP^ GTGCGGCGACAGCAGAAC--AGC**GGG**ACGATAGGCTGCAG PCR

3 CIGAR^eGFP^ GTGCGGCGACAGCAGAAC-----**GGG**ACGATAGGCTGCAG PCR

4 Recombination GTGCCCCGA-----------GAC**GGG**ACGATAGGCTGCAG PCR

5 CIGAR^eGFP^ GTGCGGCGACAGCAGAA----GC**GGG**ACGATAGGCTGCAG PCR

6 Recombination GTGCCCCGAGACAAGCACCGGGAC**GGG**ACGATAGGCTGCAG PCR

7 CIGAR^mCherry^ GTGCCCC-AGACAAGCAC-AGAC**GGG**ACGATAGGCTGCAG PCR

**8** CIGAR^mCherry^ GTGCCCCGAGACAAGCACCTGAC**GGG**ACGATAGGCTGCAG PCR

**9** CIGAR^mCherry^ GTGCCCCGAGACAAGCACCTGAC**GGG**ACGATAGGCTGCAG PCR

10 Recombination GTGCGGCGACAGCAGCGGGACAGC**GGG**ACGATAGGCTGCAG PCR

**11** CIGAR^mCherry^ GTGCCCCGAGACAAGCACCTGAC**GGG**ACGATAGGCTGCAG PCR

**12** CIGAR^mCherry^ GTGCCCCGAGACAAGCACCTGAC**GGG**ACGATAGGCTGCAG PCR

**13** CIGAR^eGFP^ GTGCGGCGACAGCAGAACGTAGC**GGG**ACGATAGGCTGCAG PCR

14 CIGAR^mCherry^ GTGCCCCGAGACAAGCACC-GAC**GGG**ACGATAGGCTGCAG PCR

**15** CIGAR^eGFP^ GTGCGGCGACAGCAGAACGTAGC**GGG**ACGATAGGCTGCAG PCR

16 Recombination GTGCCCCGAGACAAGC-----AC**GGG**ACGATAGGCTGCAG PCR

17 Recombination GTGCCCCGAGACAAGCACCTGAC**GGG**ACGATAGGCTGCAG PCR

18 CIGAR^mCherry^ GTGCCCCGA-----------GAC**GGG**ACGATAGGCTGCAG PCR

19 CIGAR^mCherry^ GTGCCCCGAGACAAGC-----AC**GGG**ACGATAGGCTGCAG PCR

20 CIGAR^mCherry^ GTGCCCCGAGACAAGC-----AC**GGG**ACGATAGGCTGCAG PCR

**21** CIGAR^mCherry^ GTGCCCCGAGACAAGCACCTGAC**GGG**ACGATAGGCTGCAG PCR

22 Recombination GTGCGGCGACAGCAGAACGTAGC**GGG**ACGATAGGCTGCAG PCR

**23** CIGAR^mCherry^ GTGCCCCGAGACAAGCACCTGAC**GGG**ACGATAGGCTGCAG PCR

24 Recombination GTGCGGCGACAGCAGAACG----**GGG**ACGATAGGCTGCAG PCR

**25** CIGAR^mCherry^ GTGCCCCGAGACAAGCACCTGAC**GGG**ACGATAGGCTGCAG PCR

26 CIGAR^eGFP^ GTGCGGCGACAGCAGAACGGGAGC**GGG**ACGATAGGCTGCAG PCR

27 Recombination GTGCCCCGACACAAGCAC--GAC**GGG**ACGATAGGCTGCAG PCR

28 Recombination GTGCCCCGA-----------GAC**GGG**ACGATAGGCTGCAG PCR

29 Recombination GTGCCCCGAGACAAGCACCTGAC**GGG**ACGATAGGCTGCAG PCR

30 Recombination GTGCCCCGAGACAAGCACCTGAC**GGG**ACGATAGGCTGCAG PCR

**31** CIGAR^eGFP^ GTGCGGCGACAGCAGAACGTAGC**GGG**ACGATAGGCTGCAG PCR

32 Recombination GTGCGGCGACAGCAGAA-----C**GGG**ACGATAGGCTGCAG PCR

**33** CIGAR^mCherry^ GTGCCCCGAGACAAGCACCTGAC**GGG**ACGATAGGCTGCAG PCR

34 CIGAR^mCherry^ GTGCCCCGAGACAAGC-----AC**GGG**ACGATAGGCTGCAG PCR

35 CIGAR^mCherry^ GTGCCCCGA-----------GAC**GGG**ACGATAGGCTGCAG PCR

**36** CIGAR^mCherry^ GTGCCCCGAGACAAGCACCTGAC**GGG**ACGATAGGCTGCAG PCR

**37** CIGAR^mCherry^ GTGCCCCGAGACAAGCACCTGAC**GGG**ACGATAGGCTGCAG PCR

**38** CIGAR^mCherry^ GTGCCCCGAGACAAGCACCTGAC**GGG**ACGATAGGCTGCAG PCR

39 CIGAR^eGFP^ GTGCGGCGACAGCAGAA-----C**GGG**ACGATAGGCTGCAG PCR

40 CIGAR^eGFP^ GTGCGGCGACAGCAGAA-----C**GGG**ACGATAGGCTGCAG PCR

**41** CIGAR^eGFP^ GTGCGGCGACAGCAGAACGTAGC**GGG**ACGATAGGCTGCAG PCR

42 CIGAR^mCherry^ GTGCCCCGAGACAAGC-----AC**GGG**ACGATAGGCTGCAG PCR

43 Recombination GTGCGGCGACAGCAGAACGGCGACAGC**GGG**ACGATAGGCTGCAG PCR

**44** CIGAR^mCherry^ GTGCCCCGAGACAAGCACCTGAC**GGG**ACGATAGGCTGCAG PCR

**45** CIGAR^mCherry^ GTGCCCCGAGACAAGCACCTGAC**GGG**ACGATAGGCTGCAG PCR

46 CIGAR^mCherry^ GTGCCCCGAGACAAGCAC--GAC**GGG**ACGATAGGCTGCAG PCR

47 CIGAR^eGFP^ GTGCGGCGACAGCAGAA-----C**GGG**ACGATAGGCTGCAG PCR

48 CIGAR^eGFP^ GTGCGGCGACAGCAG-----AGC**GGG**ACGATAGGCTGCAG PCR

49 CIGAR^mCherry^ GTGCCCCGAGACAAGCA--NGAC**GGG**ACGATAGGCTGCAG PCR

**50** CIGAR^mCherry^ GTGCCCCGAGACAAGCACCTGAC**GGG**ACGATAGGCTGCAG PCR

**51** CIGAR^eGFP^ GTGCGGCGACAGCAGAACGTAGC**GGG**ACGATAGGCTGCAG PCR

52 Recombination GTGCCCCGA-----------GAC**GGG**ACGATAGGCTGCAG PCR

53 Recombination GTGCCCCGAGACAAGCACCTGAC**GGG**ACGATAGGCTGCAG PCR

**54** CIGAR^eGFP^ GTGCGGCGACAGCAGAACGTAGC**GGG**ACGATAGGCTGCAG PCR

**55** CIGAR^eGFP^ GTGCGGCGACAGCAGAACGTAGC**GGG**ACGATAGGCTGCNN PCR

56 Recombination GTGCGGCGACAGCAGAACGACAGC**GGG**ACGATAGGCTGCAG PCR

**57** CIGAR^mCherry^ GTGCCCCGAGACAAGCACCTGAC**GGG**ACGATAGGCTGCAG PCR

58 CIGAR^mCherry^ GTGCCCCGAGACAAGC-----AC**GGG**ACGATAGGCTGCAG PCR

69 Recombination GTGCGGCGACAGCAGAACGTANC**GGG**ACGATAGGCTGCAN PCR

**60** CIGAR^eGFP^ GTGCGGCGACAGCAGAACGTAGC**GGG**ACGATAGGCTGCAG PCR

61 CIGAR^mCherry^ GTGCCCCGA-----------GAC**GGG**ACGATAGGCTGCAG PCR

62 CIGAR^eGFP^ GTGCGGCGACAGCAGAACGGACGGAGC**GGG**ACGATAGGCTGCAG PCR

63 Recombination GTGCGGCGACAGCAGAACGTAGC**GGG**ACGATAGGCTGCAG PCR

64 ? N---120bp indel----------**G**ACGATAGGCTGCAG PCR

65 Recombination GTGCCCCGAGACAAGCACCTGAC**GGG**ACGATAGGCTGCAG PCR

**66** CIGAR^eGFP^ GTGCGGCGACAGCAGAACGTAGC**GGG**ACGATAGGCTGCAG PCR

67 Recombination GTGCCCCGAGACAAGCACCTGAC**GGG**ACGATAGGCTGCAG PCR

**68** CIGAR^eGFP^ GTGCGGCGACAGCAGAACGTAGC**GGG**ACGATAGGCTGCAG PCR

69 Recombination GTGCCCCGAGACAAGCACCTGAC**GGG**ACGATAGGCTGCAG PCR

**70** CIGAR^mCherry^ GTGCCCCGAGACAAGCACCTGAC**GGG**ACGATAGGCTGCAG PCR

**71** CIGAR^mCherry^ GTGCCCCGAGACAAGCACCTGAC**GGG**ACGATAGGCTGCAG PCR

**72** CIGAR^mCherry^ GTGCCCCGAGACAAGCACCTGAC**GGG**ACGATAGGCTGCAG PCR

73 CIGAR^mCherry^ GTGCCCCGA-----------GAC**GGG**ACGATAGGCTGCAG PCR

74 CIGAR^mCherry^ GTGCCCCGA-----------GAC**GGG**ACGATAGGCTGCAG PCR

75 CIGAR^mCherry^ GTGCCCCGAGACAAGCACCAAGAC**GGG**ACGATAGGCTGCAG PCR

**76** CIGAR^mCherry^  GTGCCCCGAGACAAGCACCTGAC**GGG**ACGATAGGCTGCAG PCR

**77** CIGAR^mCherry^ GTGCCCCGAGACAAGCACCTGAC**GGG**ACGATAGGCTGCAG PCR

**78** CIGAR^mCherry^ GTGCCCCGAGACAAGCACCTGAC**GGG**ACGATAGGCTGCAG PCR

79 Recombination GTGCCCCGAGACAAGC-----AC**GGG**ACGATAGGCTGCAG PCR

80 Recombination GTGCCCCGA-----------GAC**GGG**ACGATAGGCTGCAG PCR

**81** CIGAR^mCherry^ GTGCCCCGAGACAAGCACCTGAC**GGG**ACGATAGGCTGCAG PCR

82 Recombination GTGCCCCGA-----------GAC**GGG**ACGATAGGCTGCAG PCR

83 CIGAR^mCherry^ N-27bp indel--------GAC**GGG**ACGATAGGCTGCAG PCR

84 Recombination GTGCCCCGA-----------GAC**GGG**ACGATAGGCTGCAG PCR

**Total readable sequences: 84**

**Recombination events: 26 (11 without indels; 15 with indels)**

**Total indels: 41 (this number includes recombinations with indels and larger indels)**

**Indels without recombination: 26**

**Large indels: 2 (cannot be inferred if recombined or not)**

**Un-CRISPRed CIGARs: 32**

**Unreadable or mixed sequences: 20**
