# Supplementary material for: CRISPR-induced double-strand breaks trigger recombination between homologous chromosome arms
Source: Life Sci Alliance. 2019 Jun 13;2(3):e201800267. doi: 10.26508/lsa.201800267 (PMC6587125; doi:10.26508/lsa.201800267)
Supplement: Supplementary file 2 [file LSA-2018-00267_TableS2.docx]

| **Table S2** | |  |  |  |  |  |  |  |  |  |  |  |
| --- | --- | --- | --- | --- | --- | --- | --- | --- | --- | --- | --- | --- |
| **Recombination on 4th chromosome; nosCas9; pCFD5-F1; CIGAR^eGFP102F^/CIGAR^mCherry102F^ x yw** | | | | | | | | | | | |  |
|  |  |  |  |  |  |  |  |  |  |  |  |  |
| **Sequences of 96-well plate1** | | | |  |  |  |  |  |  |  |  |  |
| 1 | 1 | 1 | fA | m2 | m2 | **m3** | f4 | f5 | m7 |  |  |  |
| **1** | 1 | 1 | **mA** | f2 | **m2** | f4 | f4 | **f5** | **mA** |  |  |  |
| **1** | 1 | 1 | mA | **f2** | **m2** | **f4** | m4 | **f5** | f1 |  |  |  |
| **1** | 1 | 1 | mA | f2 | **m2** | f4 | m4 | f5 | m1 |  |  |  |
| 1 | 1 | **mA** | fB | **f2** | m2 | f4 | m4 | m5 |  |  |  |  |
| 1 | 1 | mA | fB | **f2** | f3 | f4 | **m4** | m5 |  |  |  |  |
| 1 | 1 | **mA** | fB | m2 | f3 | **f4** | f4 | f6 |  |  |  |  |
| **1** | 1 | mA | mB | m2 | f3 | f4 | f4 | f7 |  |  |  |  |
|  |  |  |  |  |  |  |  |  |  |  |  |  |
|  |  |  |  |  |  |  |  |  |  |  |  |  |
|  |  |  |  |  |  |  |  |  |  |  |  |  |
| **Sequencesof 96-well plate2** | | | |  |  |  |  |  |  |  |  |  |
| **w12** | m12 | f5 | m5 | m5 | f7 | f14 | **f15** | m15 | **f6** | m6 | f5 |  |
| w12 | m12 | f5 | **m5** | **m5** | f7 | **f14** | f15 | m15 | f6 | m6 | **m6** |  |
| w12 | **m12** | f5 | **m5** | m5 | f7 | f14 | f15 | m15 | f6 | m6 | f7 |  |
| **w12** | m12 | f5 | m5 | m5 | f7 | f14 | f15 | **m15** | f6 | m6 | m12 |  |
| w12 | m12 | m5 | m5 | m5 | m7 | **f14** | m15 | f6 | **f6** | **m6** | **f14** |  |
| **w12** | m12 | m5 | m5 | f7 | m7 | f15 | m15 | f6 | f6 | **f13** | m15 |  |
| **m12** | **m12** | m5 | **m5** | f7 | m7 | f15 | m15 | f6 | m6 | m13 | f13 |  |
| **m12** | f5 | **m5** | m5 | f7 | m7 | f15 | m15 | f6 | m6 | m13 | f13 |  |
|  |  |  |  |  |  |  |  |  |  |  |  |  |
|  |  |  |  |  |  |  |  |  |  |  |  |  |
|  |  |  |  |  |  |  |  |  |  |  |  |  |
| m=F1 male analyzed | | |  |  |  |  |  |  |  |  |  |  |
| f=F1 female analyzed | | |  |  |  |  |  |  |  |  |  |  |
| **Bold letters indicates a recombinant. Grey shaded are "without indels", blue shaded harbor NHEJ generated indel.** | | | | | | | | | | | | |
| All picked F1 animals were pCFD5-F1 negative. | | | | |  |  |  |  |  |  |  |  |
| No PCR product or unreadable/mixed seq in orange | | | | |  |  |  |  |  |  |  |  |
| For the picked F1 flies from cross 1, the sex was not determined | | | | | | |  |  |  |  |  |  |
|  |  |  |  |  |  |  |  |  |  |  |  |  |
| **cross #** | **GO** | **Rec.** | **Info** |  |  |  |  |  |  |  |  |  |
| A | male | yes |  |  |  |  |  |  |  |  |  |  |
| B | male | no | only 4 animals tested | | |  |  |  |  |  |  |  |
| 1 | male | yes |  |  |  |  |  |  |  |  |  |  |
| 2 | male | yes |  |  |  |  |  |  |  |  |  |  |
| 3 | male | yes | wt seq only | |  |  |  |  |  |  |  |  |
| 4 | male | yes | wt seq only | |  |  |  |  |  |  |  |  |
| 5 | female | yes |  |  |  |  |  |  |  |  |  |  |
| 6 | male | yes |  |  |  |  |  |  |  |  |  |  |
| 7 | female | no | total of 13 animals tested | | |  |  |  |  |  |  |  |
| 8 | female | not tested | |  |  |  |  |  |  |  |  |  |
| 9 | male | not tested | |  |  |  |  |  |  |  |  |  |
| 10 | male | not tested | |  |  |  |  |  |  |  |  |  |
| 11 | male | not tested | |  |  |  |  |  |  |  |  |  |
| 12 | male | yes |  |  |  |  |  |  |  |  |  |  |
| 13 | female | yes | wt seq only | |  |  |  |  |  |  |  |  |
| 14 | female | yes |  |  |  |  |  |  |  |  |  |  |
| 15 | male | yes |  |  |  |  |  |  |  |  |  |  |
| Last animal plate 2 (# 96 cross number unknown) | | | | | |  |  |  |  |  |  |  |
