# Supplementary material for: CRISPR-induced double-strand breaks trigger recombination between homologous chromosome arms
Source: Life Sci Alliance. 2019 Jun 13;2(3):e201800267. doi: 10.26508/lsa.201800267 (PMC6587125; doi:10.26508/lsa.201800267)
Supplement: Supplementary file 3 [file LSA-2018-00267_TableS3.docx]

**Table S3:** Shown are the readable sequences (n=156) for recombination on the 4^th^ chromosome (n_total_=172); molecular analysis of the target site. Deleted bp shown as: –

Inserted/exchanged bp shown as: N

Bold, grey sequence numbers show unrearranged (un-CRISPRed) CIGAR reporters.

To facilitate the detection of recombination, the first four nucleotides of the shifter of CIGAR^eGFP^ (CGGC) and CIGAR^mCherry^ (CCCC) are highlighted. The **PAM** site is shown in bold letters. In addition, we highlight the PCR setup used to amplify the shifter sequences of CIGAR^eGFP^ (green) or CIGAR^mCherry^(red). The fwd primer anneals within the ubiquitin promoter (identical between both CIGAR variants), and the reverse primers primer are either specific for eGFP or mCherry, respectively.

The used primers for CIGAR^eGFP^: CIGAR-fwd: CAACAAAGTTGGCGTCGATA and CIGAR^eGFP^-rev: GAACTTCAGGGTCAGCTTGC.

The used primers for CIGAR^mCherry:^ CIGAR-fwd: CAACAAAGTTGGCGTCGATA and CIGAR^mCherry^ -rev: AAGCGCATGAACTCCTTGATG.

Orig CIGAR^eGFP^ GTGCGGCGACAGCAGAACGTAGC**GGG**ACGATAGGCTGCAG

|||| ||| | || || | |**|||**||||||||||||||

Orig CIGAR^mCherry^ GTGCCCCGAGACAAGCACCTGAC**GGG**ACGATAGGCTGCAG

1 Recombination GTGCGGCGACAGCAGAACGTAGC**GGG**ACGATAGGCTGCAG PCR

2 CIGAR^eGFP^ GTGCGGCGACAGCAGAACATGGTGC**GGG**ACGATAGGCTGC PCR

3 CIGAR^mCherry^ GTGCCCCGAGACAA----------------TAGGCTGCAG PCR

4 Recombination GTGCCCCGAGACAAGCACCTGAC**GGG**ACGATAGGCTGCAG PCR

**5** CIGAR^mCherry^ GTGCCCCGAGACAAGCACCTGAC**GGG**ACGGTAGGCTGCAG PCR

6 Recombination GTGCGGCGACAGCAGA----------ACGATAGGCTGCAG PCR

7 Recombination GTGCGGCGACAGCAGAACGACAGC**GGG**ACGATAGGCTGCA PCR

8 Recombination GTGCCCCGAGACAAGCACCTGAC**GGG**ACGATAGGCTGCAG PCR

9 CIGAR^eGFP^ GTGCGGCGACAGC-------AGC**GGG**ACGATAGGCTGCAG PCR

**10** CIGAR^mCherry^ GTGCCCCGAGACAAGCACCTGAC**GGG**ACGATAGGCTGCAG PCR

11 Recombination GTGCGGCGACAGCAGAACGACAGC**GGG**ACGATAGGCTGCA PCR

**12** CIGAR^mCherry^ GTGCCCCGAGACAAGCACCTGAC**GGG**ACGATAGGCTGCAG PCR

13 CIGAR^eGFP^ GTGCGGCGACAGCAGA----------ACGATAGGCTGCAG PCR

**14** CIGAR^mCherry^ GTGCCCCGAGACAAGCACCTGAC**GGG**ACGATAGGCTGCAG PCR

15 Recombination GTGCGGCGACAGCAGAACGACAGC**GGG**ACGATAGGCTGCA PCR

16 CIGAR^mCherry^ GTGCCCCGAGA-----------C**GGG**ACGATAGGCTGCAG PCR

**17** CIGAR^mCherry^ GTGCCCCGAGACAAGCACCTGAC**GGG**ACGATAGGCTGCAG PCR

18 CIGAR^mCherry^ GTGCCCCGAGACAAGCACCGAGACAAGAC**GGG**ACGATAGG PCR

19 CIGAR^eGFP^ GTGCGGCGAC----------AGC**GGG**ACGATAGGCTGCAG PCR

20 ? G-------------------------ACGGGACGATAGGC PCR

21 CIGAR^eGFP^ GTGCGGCGACAGCAG-----AGC**GGG**ACGATAGGCTGCAG PCR

**22** CIGAR^eGFP^ GTGCGGCGACAGCAGAACGTAGC**GGG**ACGATAGGCTGCAG PCR

23 Recombination GTGCGGCGACAGCAGAACGTAGC**GGG**ACGATAGGCTGCAG PCR

24 CIGAR^mCherry^ GTGCCCCGA-----------GAC**GGG**ACGATAGGCTGCAG PCR

25 Recombination GTGCCCCGAGACAAGCACCTGAC**GGG**ACGATAGGCTGCAG PCR

26 CIGAR^mCherry^ GTGCCCCGAGACAAGC---TGAC**GGG**ACGATAGGCTGCAG PCR

27 CIGAR^eGFP^ GTGCGGCGACAGC-------AGC**GGG**ACGATAGGCTGCAG PCR

**28** CIGAR^mCherry^ GTGCCCCGAGACAAGCACCTGAC**GGG**ACGATAGGCTGCAG PCR

29 Recombination GTGCGGCGACAGCAGAA-----C**GGG**ACGATAGGCTGCAG PCR

30 CIGAR^eGFP^ GTGCGGCGACAGCAGAA-----C**GGG**ACGATAGGCTGCAG PCR

**31** CIGAR^eGFP^ GTGCGGCGACAGCAGAACGTAGC**GGG**ACGATAGGCTGCAG PCR

32 Recombination GTGCCCCGAGACAAGCACCTGAC**GGG**ACGATAGGCTGCAG PCR

**33** CIGAR^mCherry^ GTGCCCCGAGACAAGCACCTGAC**GGG**ACGATAGGCTGCAG PCR

34 CIGAR^mCherry^ GTGCCCCGAGACAAG---------44bp del-------- PCR

**35** CIGAR^eGFP^ GTGCGGCGACAGCAGAACGTAGC**GGG**ACGATAGGCTGCAG PCR

36 ? N------34bp indel-----C**GGG**ACGATAGGCTGCAG PCR

37 CIGAR^eGFP^ GTGCGGCGACAGCAG-------C**GGG**ACGATAGGCTGCAG PCR

**38** CIGAR^mCherry^ GTGCCCCGAGACAAGCACCTGAC**GGG**ACGATAGGCTGCAG PCR

39 CIGAR^mCherry^ GTGCCCCGAGACAAGCAC----C**GGG**ACGATAGGCTGCAG PCR

**40** CIGAR^mCherry^ GTGCCCCGAGACAAGCACCTGAC**GGG**ACGATAGGCTGCAG PCR

41 CIGAR^eGFP^ GTGCGGCGACAGC-------AGC**GGG**ACGATAGGCTGCAG PCR

42 CIGAR^eGFP^ GTGCGGCGAC----------AGC**GGG**ACGATAGGCTGCAG PCR

**43** CIGAR^mCherry^ GTGCCCCGAGACAAGCACCTGAC**GGG**ACGATAGGCTGCAG PCR

**44** CIGAR^mCherry^ GTGCCCCGAGACAAGCACCTGAC**GGG**ACGATAGGCTGCAG PCR

**45** CIGAR^mCherry^ GTGCCCCGAGACAAGCACCTGAC**GGG**ACGATAGGCTGCAG PCR

**46** CIGAR^mCherry^ GTGCCCCGAGACAAGCACCTGAC**GGG**ACGATAGGCTGCAG PCR

47 Recombination GTGCCCCGAGACAAGCACCTGAC**GGG**ACGATAGGCTGCAG PCR

**48** CIGAR^mCherry^ GTGCCCCGAGACAAGCACCTGAC**GGG**ACGATAGGCTGCAG PCR

**49** CIGAR^eGFP^ GTGCGGCGACAGCAGAACGTAGC**GGG**ACGATAGGCTGCAG PCR

50 Recombination GTGCCCCGAGACAAGCATGTCGGGTGCCCCCGAGAC**GGG**A PCR

**51** CIGAR^mCherry^ GTGCCCCGAGACAAGCACCTGAC**GGG**ACGATAGGCTGCAG PCR

**52** CIGAR^mCherry^ GTGCCCCGAGACAAGCACCTGAC**GGG**ACGATAGGCTGCAG PCR

53 Recombination GTGCCCCGAGACAAGCACCTGAC**GGG**ACGATAGGCTGCAG PCR

54 CIGAR^mCherry^ GTGCCCCGAGACAAGCACCGGGACAGAC**GGG**ACGATAGGC PCR

55 CIGAR^mCherry^ GTGCCCCGAGACAAGCACCATGTCCCGAGAC**GGG**ACGATA PCR

56 CIGAR^eGFP^ GTGCGGCGACAGCAGACGACAGC**GGG**ACGATAGGCTGCAG PCR

**57** CIGAR^eGFP^ GTGCGGCGACAGCAGAACGTAGC**GGG**ACGATAGGCTGCAG PCR

**58** CIGAR^mCherry^ GTGCCCCGAGACAAGCACCTGAC**GGG**ACGATAGGCTGCAG PCR

59 CIGAR^eGFP^ GTGCGGCGACAGCAGAAC--AGC**GGG**ACGATAGGCTGCAG PCR

60 CIGAR^mCherry^ GTGCGGCGAC----------AGC**GGG**ACGATAGGCTGCAG PCR

**61** CIGAR^mCherry^ GTGCCCCGAGACAAGCACCTGAC**GGG**ACGATAGGCTGCAG PCR

62 Recombination GTGCCCCGAGACAAG----GGAC**GGG**ACGATAGGCTGCAG PCR

**63** CIGAR^mCherry^ GTGCCCCGAGACAAGCACCTGAC**GGG**ACGATAGGCTGCAG PCR

**64** CIGAR^mCherry^ GTGCCCCGAGACAAGCACCTGAC**GGG**ACGATAGGCTGCAG PCR

**65** CIGAR^mCherry^ GTGCCCCGAGACAAGCACCTGAC**GGG**ACGATAGGCTGCAG PCR

66 Recombination GTGCGGCGACAGCAGAAC--AGC**GGG**ACGATAGGCTGCAG PCR

**67** CIGAR^mCherry^ GTGCCCCGAGACAAGCACCTGAC**GGG**ACGATAGGCTGCAG PCR

**68** CIGAR^mCherry^ GTGCCCCGAGACAAGCACCTGAC**GGG**ACGATAGGCTGCAG PCR

69 CIGAR^mCherry^ GTGCCCCGAGACAAGCA-------------------GCAG PCR

70 Recombination GTGCCCCGAGACAAGCACCTGAC**GGG**ACGATAGGCTGCAG PCR

71 CIGAR^mCherry^ GTGCGGCGACAGCAG----------**G**ACGATAGGCTGCAG PCR

**72** CIGAR^mCherry^ GTGCCCCGAGACAAGCACCTGAC**GGG**ACGATAGGCTGCAG PCR

**73** CIGAR^mCherry^ GTGCCCCGAGACAAGCACCTGAC**GGG**ACGATAGGCTGCAG PCR

**74** CIGAR^mCherry^ GTGCCCCGAGACAAGCACCTGAC**GGG**ACGATAGGCTGCAG PCR

75 Recombination GTGCCCCGAGACAAG-----GAC**GGG**ACGATAGGCTGCAG PCR

76 Recombination GTGCCCCGAGACAAGCACCTGAC**GGG**ACGATAGGCTGCAG PCR

**77** CIGAR^mCherry^ GTGCCCCGAGACAAGCACCTGAC**GGG**ACGATAGGCTGCAG PCR

**78** CIGAR^mCherry^ GTGCCCCGAGACAAGCACCTGAC**GGG**ACGATAGGCTGCAG PCR

**79** CIGAR^mCherry^ GTGCCCCGAGACAAGCACCTGAC**GGG**ACGATAGGCTGCAG PCR

80 Recombination GTGCCCCGAGACAAGCACCTGAC**GGG**ACGATAGGCTGCAG PCR

81 CIGAR^eGFP^ GTGCGGCGACAGCAGAACG-AGC**GGG**ACGATAGGCTGCAG PCR

82 ? GT-------------------GC**GGG**ACGATAGGCTGCAG PCR

83 Recombination GTGCCCCGAGACAAGCACGAGAC**GGG**ACGATAGGCTGCAG PCR

**84** CIGAR^mCherry^ GTGCCCCGAGACAAGCACCTGAC**GGG**ACGATAGGCTGCAG PCR

85 CIGAR^eGFP^ GTGCGGCGACAGCAGA----------ACGATAGGCTGCAG PCR

**86** CIGAR^mCherry^ GTGCCCCGAGACAAGCACCTGAC**GGG**ACGATAGGCTGCAG PCR

87 CIGAR^mCherry^ GTGCCCCGAGACAAGCAC-TGAC**GGG**ACGATAGGCTGCAG PCR

88 Recombination GTGCCCCGA-----------GAC**GGG**ACGATAGGCTGCAG PCR

89 Recombination GTGCCCCGAGACAA------GAC**GGG**ACGATAGGCTGCAG PCR

90 Recombination GTGCGGCGACAGC-------AGC**GGG**ACGATAGGCTGCAG PCR

91 CIGAR^mCherry^ GTGCCCCGA----------------**G**ACGATAGGCTGCAG PCR

**92** CIGAR^mCherry^ GTGCCCCGAGACAAGCACCTGAC**GGG**ACGATAGGCTGCAG PCR

93 Recombination GTGCGGCGACAGC----------**GGG**ACGATAGGCTGCAG PCR

94 CIGAR^eGFP^ GTGCGGCGACAGCAGAACGGGACGATA**GGG**ACGATAGGCT PCR

95 CIGAR^eGFP^ GTGCGGCGACAGCAGA----------ACGATAGGCTGCAG PCR

96 CIGAR^mCherry^ GTGCCCCGAGACAAGCAC-TGAC**GGG**ACGATAGGCTGCAG PCR

**97** CIGAR^mCherry^ GTGCCCCGAGACAAGCACCTGAC**GGG**ACGATAGGCTGCAG PCR

98 CIGAR^mCherry^ GTGCCCCGAGACAAGGACGTCCCGAGAC**GGG**ACGATAGGC PCR

**99** CIGAR^mCherry^ GTGCCCCGAGACAAGCACCTGAC**GGG**ACGATAGGCTGCAG PCR

100 CIGAR^mCherry^ GTGCCCCGAGACAAG-ACAAGAC**GGG**ACGATAGGCTGCAG PCR

101 CIGAR^eGFP^ GTGCGGCGACAGCAGA----------ACGATAGGCTGCAG PCR

**102** CIGAR^mCherry^ GTGCCCCGAGACAAGCACCTGAC**GGG**ACGATAGGCTGCAG PCR

**103** CIGAR^mCherry^ GTGCCCCGAGACAAGCACCTGAC**GGG**ACGATAGGCTGCAG PCR

104 ? GTG-------------------C**GGG**ACGATAGGCTGCAG PCR

**105** CIGAR^mCherry^ GTGCCCCGAGACAAGCACCTGAC**GGG**ACGATAGGCTGCAG PCR

106 Recombination GTGCGGCGACAGC-------AGC**GGG**ACGATAGGCTGCAG PCR

107 CIGAR^eGFP^ GTGCGGCGACAGCAG-----AGC**GGG**ACGATAGGCTGCAG PCR

108 Recombination GTGCCCCGAGACAAGCACCTGAC**GGG**ACGATAGGCTGCAG PCR

109 CIGAR^eGFP^ GTGCGGCGACAGCAGAACG-AGC**GGG**ACGATAGGCTGCAG PCR

110 CIGAR^eGFP^ GTGCGGCGACAGCAGAACG-AGCGGGACGATAGGCTGCAG PCR

111 Recombination GTGCGGCGACAGCAGAACGTCGGGAACAGAACAGC**GGG**AC PCR

112 CIGAR^eGFP^ GTGCGGCGACAGCAGAACG-AGC**GGG**ACGATAGGCTGCAG PCR

113 CIGAR^mCherry^ GTGCCCCGAGACAAGCACGGGAC**GGG**ACGATAGGCTGCAG PCR

**114** CIGAR^eGFP^ GTGCGGCGACAGCAGAACGTAGC**GGG**ACGATAGGCTGCAG PCR

115 CIGAR^eGFP^ GTGCGGCGACAGCA-----------**G**ACGATAGGCTGCAG PCR

116 ? N------------------------**G**ACGATAGGCTGCAG PCR

117 CIGAR^eGFP^ GTGCGGCGACAGCAGAACGACAGC**GGG**ACGATAGGCTGCA PCR

118 CIGAR^eGFP^ GTGCGGCGACAGCAGACAGCAGACAGCAGACAGC**GGG**ACG PCR

**119** CIGAR^mCherry^ GTGCCCCGAGACAAGCACCTGAC**GGG**ACGATAGGCTGCAG PCR

120 Recombination GTGCCCCGAGACAAGCACCTGAC**GGG**ACGATAGGCTGCAG PCR

121 ? N------------------------**G**ACGATAGGCTGCAG PCR

122 Recombination GTGCCCCGAGACAAGCAC--G--**GGG**-CGATAGGCTGCAG PCR

123 Recombination GTGCCCCGAGACAAGCACCTGAC**GGG**ACGATAGGCTGCAG PCR

124 CIGAR^eGFP^ GTGCGGCGACAGCAGAACG-AGC**GGG**ACGATAGGCTGCAG PCR

125 CIGAR^mCherry^ GTGCCCCGAGACAAGCAC--GAC**GGG**ACGATAGGCTGCAG PCR

126 Recombination GTGCCCCGAGACAAGCACCA-AT**G**A**G**ACGATAGGCTGCAG PCR

127 Recombination GTGCCCCGAGACAAGCACCTGAC**GGG**ACGATAGGCTGCAG PCR

128 Recombination GTGCCCCGAGACAAGCACCTGAC**GGG**ACGATAGGCTGCAG PCR

**129** CIGAR^mCherry^ GTGCCCCGAGACAAGCACCTGAC**GGG**ACGATAGGCTGCAG PCR

130 CIGAR^eGFP^ GTGCGGCGACAGCA------AGC**GGG**ACGATAGGCTGCAG PCR

**131** CIGAR^mCherry^ GTGCCCCGAGACAAGCACCTGAC**GGG**ACGATAGGCTGCAG PCR

132 CIGAR^mCherry^ GTGCCCCGAGCCAAGCACCTGAC**GGG**ACGATAGGCTGCAG PCR

133 Recombination GTGCGGCGACAGCAGAACGTAGC**GGG**ACGATAGGCTGCAG PCR

134 CIGAR^mCherry^ GTGCCCCGAGACAAGCAC--GAC**GGG**ACGATAGGCTGCAG PCR

135 Recombination GTGCCCCGAGACAAGCACCTGAC**GGG**ACGATAGGCTGCAG PCR

136 CIGAR^eGFP^ GTGCGGCGACAGCAGAAC-----**GGG**ACGATAGGCTGCAG PCR

137 CIGAR^eGFP^ GTGCGGCGACAGCAGAAC-TAGC**GGG**ACGATAGGCTGCAG PCR

138 Recombination GTGCCCCGAGACAAGCACCTGAC**GGG**ACGATAGGCTGCAG PCR

139 CIGAR^mCherry^ GTGCCCCGAGACAAGCACC-----**GG**ACGATAGGCTGCAG PCR

**140** CIGAR^mCherry^ GTGCCCCGAGACAAGCACCTGAC**GGG**ACGATAGGCTGCAG PCR

141 CIGAR^eGFP^ GTGCGGCGAC----------AGC**GGG**ACGATAGGCTGCAG PCR

142 CIGAR^mCherry^ GTGCCCCGA-----------GAC**GGG**ACGATAGGCTGCAG PCR

143 Recombination GTGCCCCGAGACAAGCACCTGAC**GGG**ACGATAGGCTGCAG PCR

144 CIGAR^eGFP^ GTGCGGCGACAGCAGA----------ACGATAGGCTGCAG PCR

145 ? GTG-TCC----C--G-A---GAC**GGG**ACGATAGGCTGCAG PCR

146 Recombination GTGCCCCGAGACAAGCACCTGAC**GGG**ACGATAGGCTGCAG PCR

147 Recombination GTGCGGCGACAGCAGAACG-AGC**GGG**ACGATAGGCTGCAG PCR

**148** CIGAR^mCherry^ GTGCCCCGAGACAAGCACCTGAC**GGG**ACGATAGGCTGCAG PCR

**149** CIGAR^mCherry^ GTGCCCCGAGACAAGCACCTGAC**GGG**ACGATAGGCTGCAG PCR

150 CIGAR^mCherry^ GTGCCCCGAGACAAGCAC-TGAC**GGG**ACGATAGGCTGCAG PCR

**151** CIGAR^mCherry^ GTGCCCCGAGACAAGCACCTGAC**GGG**ACGATAGGCTGCAG PCR

152 CIGAR^mCherry^ GTGCCCCGA-----------GAC**GGG**ACGATAGGCTGCAG PCR

**153** CIGAR^eGFP^ GTGCGGCGACAGCAGAACGTAGC**GGG**ACGATAGGCTGCAG PCR

154 Recombination GTGCGGCGACAGCAGACGACAGACAGC**GGG**ACGATAGGCT PCR

155 CIGAR^eGFP^ GTGCGGCGACAGC-------AGC**GGG**ACGATAGGCTGCAG PCR

156 ? N-174bp del-from promoter into happy-linker PCR (shorter band in PCR)

**Total readable sequences: 156**

**Recombination events: 41 (21 without indels; 20 with indels)**

**Total indels: 84 (this number includes recombinations with indels and larger indels)**

**Indels without recombination: 64**

**Large indels: 8 (cannot be inferred if recombined or not)**

**Un-CRISPRed CIGARs: 51**

**Unreadable or mixed sequences: 16**
