# Supplementary material for: CRISPR-induced double-strand breaks trigger recombination between homologous chromosome arms
Source: Life Sci Alliance. 2019 Jun 13;2(3):e201800267. doi: 10.26508/lsa.201800267 (PMC6587125; doi:10.26508/lsa.201800267)
Supplement: Supplementary file 4 [file LSA-2018-00267_TableS4.docx]

**Table S4:**

Recombination between two visible markers.

Recombination is induced between w+ (CIGAR^mCherry,w+^ at ZH attP 102F) and sv^spa-pol^ (on chromosome Dp(1;4)1021, y+(left arm of 4th chromosome) sv^spa-pol^).

CTGTTGATAAGCACGCAATC is the sgRNA-3 used for the experiment.

Direct injection of sgRNA-3/Cas9 complexes

**PCR Primers**

For sequencing the primer **AACTACCATCAGGACTTTCAAG** was used

PCR product (**using primers**):

**AACTACCATCAGGACTTTCAAG**TAATTTAAATAGGCTGAACCTTTTCGGTCTGAAACTTGTGAATTGCACACTATATGTATTTTAAAAGGCAAATGAAATGTTAAATCTACATTAAAATTGATGTTATTTTTGAATAAAAATTCAAGATTTGTAAACAATATTTCCGCATCTTTCGCATCCTTTTTTAAGTTCCAAACATTCCTGATCTGCTAAGATAGGTTAAAGTATTTCGGGGAATCTTCAACTTTA**CTGTTGATAAGCACGCAATCGGG**ATATTAGACTTCTGCAGTTGCTTGCAATTTTTTAAGCCTTCACAATTATATTTATATTTTGAACCATTTTTTGATATTATAAATTAAACTTATTCATGTATTAATACTATTTATTAATCGTCTTTAAGTAAAGAATGAGCAATTATGCTCATACATACTTAAAAAAAAAACCATGTGTTACCAAACTTAGGGAAGTGCGTGGAAGACGGATATAAAGCAAAGTGACGTTATACATGATTTTACATTTACATTAAATTCTTATATGACAATTTATGTTTTTA**GAGTCGTAGGCGTTATATAGTT**

**Sequences of the target sites of the recovered recombinants:**

Genotype of the recovered animals (lines):

1-1: yw; CIGAR^mCherry,102F,^**^w+,^** **sv^spa-pol^** / Dp(1:4)1021, y+, sv^spa-pol^

52-1: yw; Dp(1:4)1021, y+ , **sv+** / Dp(1:4)1021, y+, sv^spa-pol^ **(**🡪 **w^-^)**

Original GGGGAATCTTCAACTTTACTGTTGATAAGCACGCAATC**GGG**ATATTAGACTTCTGCAG

sgRNA-3 PAM

line:

1-1 GGGGAATCTTCAACTTTACTGTTGATAAGCACGCAATCGGGATATTAGACTTCTGCAG

7-1 GGGGAATCTTCAACTTTACTGTTGATAAGCACGCAATCGGGATATTAGACTTCTGCAG

18-1 GGGGAATCTTCAACTTTACTGTTGATAAGCACGCAATCGGGATATTAGACTTCTGCAG

41-1 GGGGAATCTTCAACTTTACTGTTGATAAGCACGCAATCGGGATATTAGACTTCTGCAG

46-1 GGGGAATCTTCAACTTTACTGTTGATAAGCACGCAATCGGGATATTAGACTTCTGCAG

52-1 GGGGAATCTTCAACTTTACTGTTGATAAGCACGCAATCGGGATATTAGACTTCTGCAG

53-1 GGGGAATCTTCAACTTTACTGTTGATAAGCACGCAATCGGGATATTAGACTTCTGCAG

59-1 GGGGAATCTTCAACTTTACTGTTGATAAGCACGCAATCGGGATATTAGACTTCTGCAG

61-2 GGGGAATCTTCAACTTTACTGTTGATAAGCACGCAATCGGGATATTAGACTTCTGCAG

70-1 GGGGAATCTTCAACTTTACTGTTGATAAGCACGCAATCGGGATATTAGACTTCTGCAG

75-1 GGGGAATCTTCAACTTTACTGTTGATAAGCACGCAATCGGGATATTAGACTTCTGCAG

85-1 GGGGAATCTTCAACTTTACTGTTGATAAGCACGCAATCGGGATATTAGACTTCTGCAG

85-2* GGGGAATCTTCAACTTTACTGTTGATAAGCA------CGGGATATTAGACTTCTGCAG

85-3* GGGGAATCTTCAACTTTACTGTTGATAAGCA------CGGGATATTAGACTTCTGCAG

91-1 GGGGAATCTTCAACTTTACTGTTGATAAGCACGCAATCGGGATATTAGACTTCTGCAG

93-1 GGGGAATCTTCAACTTTACTGTTGATAAGCACGCAATCGGGATATTAGACTTCTGCAG

108-2 GGGGAATCTTCAACTTTACTGTTGATAAGCACGCAATCGGGATATTAGACTTCTGCAG

112-1 GGGGAATCTTCAACTTTACTGTTGATAAGCACGCAATCGGGATATTAGACTTCTGCAG

115-1 GGGGAATCTTCAACTTTACTGTTGATAAGCACGCAATCGGGATATTAGACTTCTGCAG

115-2 GGGGAATCTTCAACTTTACTGTTGATAAGCACGCAATCGGGATATTAGACTTCTGCAG

117-1 GGGGAATCTTCAACTTTACTGTTGATAAGCACGCAATCGGGATATTAGACTTCTGCAG

Except for the recombinants 115-1 and 115-2, the recombination events monitored are truly independent

* Sequences shown below.

Sequence trace 85-2:


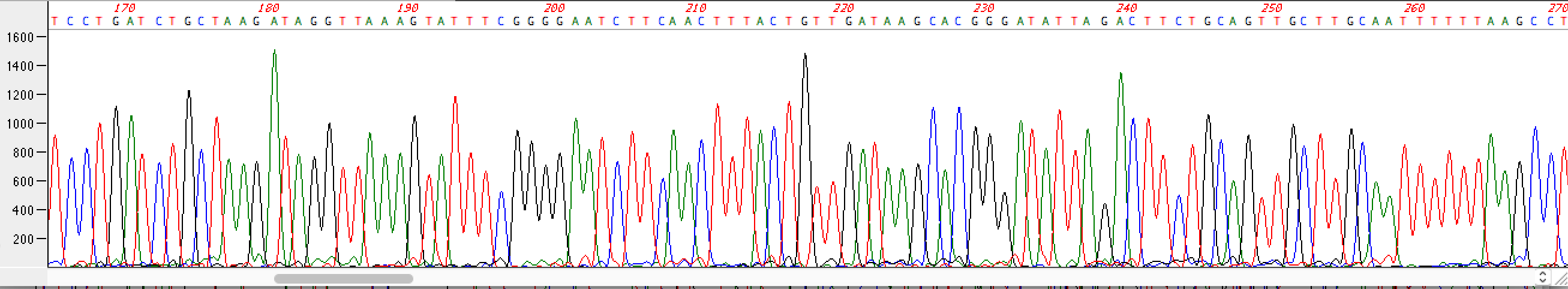


Sequence trace 85-3:


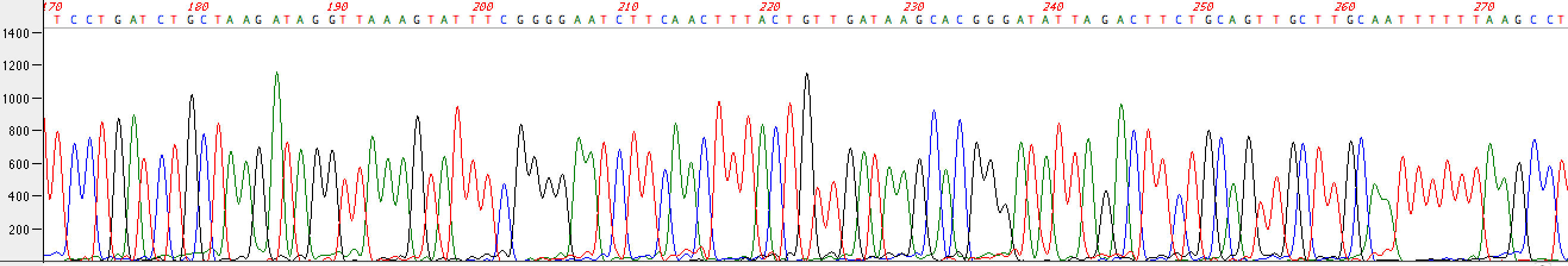


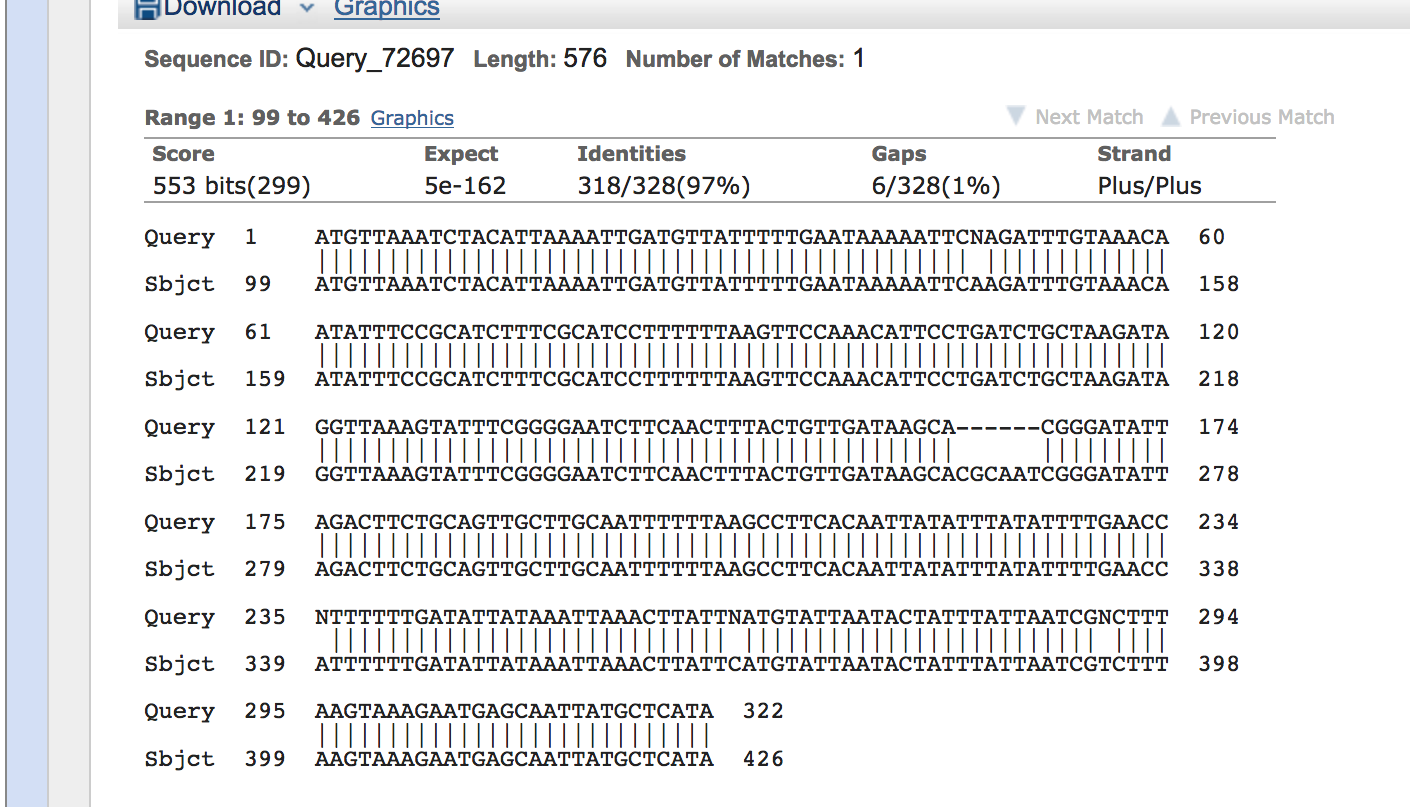


Sequence alignment of an unrearranged target site and target site with 6bp indel (using blastn: https://blast.ncbi.nlm.nih.gov )
