# Supplementary material for: CRISPR-induced double-strand breaks trigger recombination between homologous chromosome arms
Source: Life Sci Alliance. 2019 Jun 13;2(3):e201800267. doi: 10.26508/lsa.201800267 (PMC6587125; doi:10.26508/lsa.201800267)
Supplement: Supplementary file 5 [file LSA-2018-00267_TableS5.docx]

| **Table S5:** | | | | |  |  | |  | | |  |  |
| --- | --- | --- | --- | --- | --- | --- | --- | --- | --- | --- | --- | --- |
| List of recombinants recovered from yw; CIGAR^mCherry 102F, w+^/Dp(1021) y^+^, sv^spa-pol^ injected with Cas9 | | | | | | | | | | | |  |
| PEV = Position Effect Variegation | | | | | |  | |  | | |  |  |
| yw; CIGAR^mCherry 102F, w+^, sv^spa-pol^ / Dp(1:4)1021, y^+^, sv^spa-pol^ **Phenotype red, rough eyes** (TR-A; Fig 3E) | | | | | | | | | | | |  |
| yw; Dp(1:4)1021, y^+^ , sv^+^ / Dp(1:4)1021, y^+^, sv^spa-pol^ **Phenotype white, smooth eyes** (TR-B; Fig 3E) | | | | |  |  | |  | | |  |  |
|  | | | | |  |  | |  | | |  |  |
| **Name** | **homozygous viable** | **Viable over Ci^D^, sv^spa-pol^** | **Viable over sv^Δ122^** | **Genotype of recovered animal** | | | **Sequence at CRISPR target site homo-zygous animals only** | | |  |  |  |
|  |  |  |  |  | | |  | | |  |  |  |
|  |  |  |  |  | | |  | | |  |  |  |
|  |  |  |  |  | | |  | | |  |  |  |
| 1-1 | yes | yes | yes | yw; CIGAR^mCherry,102F^, w^+^, sv^spa-pol^ / Dp(1;4)1021, y^+^, sv^spa-pol^ | | | wild-type no indel | | |  |  |  |
| 7-1 | yes | n.d. | n.d. | yw; CIGAR^mCherry,102F^, w^+^, sv^spa-pol^ / Dp(1;4)1021, y^+^, sv^spa-pol^ | | | wild-type no indel | | |  |  |  |
| 18-1 | yes | yes | yes | yw; CIGAR^mCherry,102F^, w^+^, sv^spa-pol^ / Dp(1;4)1021, y^+^, sv^spa-pol^ | | | wild-type no indel | | |  |  |  |
| 13-1 PEV | no | n.d. | n.d. | yw; CIGAR^mCherry,102F^, w^+^/ Dp(1;4)1021, y^+^, sv^spa-pol^ | | |  | | |  |  |  |
| 13-2 PEV | no | n.d. | n.d. | yw; CIGAR^mCherry,102F^, w^+^/ Dp(1;4)1021, y^+^, sv^spa-pol^ | | |  | | |  |  |  |
| 18-2 PEV | no | n.d. | n.d. | yw; CIGAR^mCherry,102F^, w^+^/ Dp(1;4)1021, y^+^, sv^spa-pol^ | | |  | | |  |  |  |
| 18-3 PEV | no | yes | no | yw; CIGAR^mCherry,102F^, w^+^/ Dp(1;4)1021, y^+^, sv^spa-pol^ | | |  | | |  |  |  |
| 18-4 PEV | no | n.d. | n.d. | yw; CIGAR^mCherry,102F^, w^+^/ Dp(1;4)1021, y^+^, sv^spa-pol^ | | |  | | |  |  |  |
| 24-1 PEV | no | n.d. | n.d. | yw; CIGAR^mCherry,102F^, w^+^/ Dp(1;4)1021, y^+^, sv^spa-pol^ | | |  | | |  |  |  |
| 29-1 PEV | no | n.d. | n.d. | yw; CIGAR^mCherry,102F^, w^+^/ Dp(1;4)1021, y^+^, sv^spa-pol^ | | |  | | |  |  |  |
| 30-1 PEV | no | n.d. | n.d. | yw; CIGAR^mCherry,102F^, w^+^/ Dp(1;4)1021, y^+^, sv^spa-pol^ | | |  | | |  |  |  |
| 32-1 PEV | no | n.d. | n.d. | yw; CIGAR^mCherry,102F^, w^+^/ Dp(1;4)1021, y^+^, sv^spa-pol^ | | |  | | |  |  |  |
| 35-1 PEV | no | n.d. | n.d. | yw; CIGAR^mCherry,102F^, w^+^/ Dp(1;4)1021, y^+^, sv^spa-pol^ | | |  | | |  |  |  |
| 38-1 PEV | no | n.d. | n.d. | yw; CIGAR^mCherry,102F^, w^+^ Dp(1;4)1021, y^+^, sv^spa-pol^ | | |  | | |  |  |  |
| 40-1 PEV | no | yes | no | yw; CIGAR^mCherry,102F^, w^+^/ Dp(1;4)1021, y^+^, sv^spa-pol^ | | |  | | |  |  |  |
| 40-2 PEV | no | n.d. | n.d. | yw; CIGAR^mCherry,102F^, w^+^/ Dp(1;4)1021, y^+^, sv^spa-pol^ | | |  | | |  |  |  |
| 41-1 | yes | yes | yes | yw; CIGAR^mCherry,102F^, w^+^, sv^spa-pol^ / Dp(1;4)1021, y^+^, sv^spa-pol^ | | | wild-type no indel | | |  |  |  |
| 42-1 PEV | no | n.d. | n.d. | yw; CIGAR^mCherry,102F^, w^+^/ Dp(1;4)1021, y^+^, sv^spa-pol^ | | |  | | |  |  |  |
| 45-1 PEV | no | n.d. | n.d. | yw; CIGAR^mCherry,102F^, w^+^/ Dp(1;4)1021, y^+^, sv^spa-pol^ | | |  | | |  |  |  |
| 46-1 | yes | yes | yes | yw; CIGAR^mCherry,102F^, w^+^, sv^spa-pol^ / Dp(1;4)1021, y^+^, sv^spa-pol^ | | | wild-type no indel | | |  |  |  |
| 49-1 PEV | no | n.d. | n.d. | yw; CIGAR^mCherry,102F^, w^+^/ Dp(1;4)1021, y^+^, sv^spa-pol^ | | |  | | |  |  |  |
| 50-1 PEV | no | n.d. | n.d. | yw; CIGAR^mCherry,102F^, w^+^/ Dp(1;4)1021, y^+^, sv^spa-pol^ | | |  | | |  |  |  |
| 51-1 PEV | no | n.d. | n.d. | yw; CIGAR^mCherry,102F^, w^+^/ Dp(1;4)1021, y^+^, sv^spa-pol^ | | |  | | |  |  |  |
| 52-1 | yes | yes | yes | yw; Dp(1:4)1021, y^+^ , sv^+^ / Dp(1:4)1021, y^+^, sv^spa-pol^ | | | wild-type no indel | | |  |  |  |
| 53-1 | yes | yes | yes | yw; CIGAR^mCherry,102F^, w^+^, sv^spa-pol^ / Dp(1;4)1021, y^+^, sv^spa-pol^ | | | wild-type no indel | | |  |  |  |
| 53-2 PEV | no | n.d. | n.d. | yw; CIGAR^mCherry,102F^, w^+^/ Dp(1;4)1021, y^+^, sv^spa-pol^ | | |  | | |  |  |  |
| 54-2 PEV | no | n.d. | n.d. | yw; CIGAR^mCherry,102F^, w^+^/ Dp(1;4)1021, y^+^, sv^spa-pol^ | | |  | | |  |  |  |
| 54-3 PEV | no | n.d. | n.d. | yw; CIGAR^mCherry,102F^, w^+^/ Dp(1;4)1021, y^+^, sv^spa-pol^ | | |  | | |  |  |  |
| 55-1 PEV | no | n.d. | n.d. | yw; CIGAR^mCherry,102F^, w^+^/ Dp(1;4)1021, y^+^, sv^spa-pol^ | | |  | | |  |  |  |
| 57-1 PEV | no | n.d. | n.d. | yw; CIGAR^mCherry,102F^, w^+^/ Dp(1;4)1021, y^+^, sv^spa-pol^ | | |  | | |  |  |  |
| 59-1 | yes | n.d. | n.d. | yw; CIGAR^mCherry,102F^, w^+^, sv^spa-pol^ / Dp(1;4)1021, y^+^, sv^spa-pol^ | | | wild-type no indel | | |  |  |  |
| 61-1 PEV | no | n.d. | n.d. | yw; CIGAR^mCherry,102F^, w^+^/ Dp(1;4)1021, y^+^, sv^spa-pol^ | | |  | | |  |  |  |
| 61-2 | yes | yes | yes | yw; CIGAR^mCherry,102F^, w^+^, sv^spa-pol^ / Dp(1;4)1021, y^+^, sv^spa-pol^ | | | wild-type no indel | | |  |  |  |
| 67-1 PEV | no | n.d. | n.d. | yw; CIGAR^mCherry,102F^, w^+^/ Dp(1;4)1021, y^+^, sv^spa-pol^ | | |  | | |  |  |  |
| 70-1 | yes | n.d. | n.d. | yw; CIGAR^mCherry,102F^, w^+^, sv^spa-pol^ / Dp(1;4)1021, y^+^, sv^spa-pol^ | | | wild-type no indel | | |  |  |  |
| 75-1 | yes | n.d. | n.d. | yw; CIGAR^mCherry,102F^, w^+^, sv^spa-pol^ / Dp(1;4)1021, y^+^, sv^spa-pol^ | | | wild-type no indel | | |  |  |  |
| 85-1 | yes | yes | yes | yw; CIGAR^mCherry,102F^, w+, sv^spa-pol^ / Dp(1;4)1021, y^+^, sv^spa-pol^ | | | wild-type no indel | | |  |  |  |
| 85-2 | yes | n.d. | n.d. | yw; CIGAR^mCherry,102F^, w^+^, sv^spa-pol^ / Dp(1;4)1021, y^+^, sv^spa-pol^ | | | **6 bp deletion** | | |  |  |  |
| 85-3 | yes | yes | yes | yw; Dp(1:4)1021, y^+^ , sv^+^ / Dp(1;4)1021, y^+^, sv^spa-pol^ | | | **6 bp deletion** | | |  |  |  |
| 91-1 | yes | n.d. | n.d. | yw; CIGAR^mCherry,102F^, w^+^, sv^spa-pol^ / Dp(1;4)1021, y^+^, sv^spa-pol^ | | | wild-type no indel | | |  |  |  |
| 92-1 PEV | no | n.d. | n.d. | yw; CIGAR^mCherry,102F^, w^+^/ Dp(1;4)1021, y^+^, sv^spa-pol^ | | |  | | |  |  |  |
| 93-1 | yes | yes | yes | yw; Dp(1:4)1021, y^+^ , sv^+^ / Dp(1;4)1021, y^+^, sv^spa-pol^ | | | wild-type no indel | | |  |  |  |
| 97-1 PEV | no | n.d. | n.d. | yw; CIGAR^mCherry,102F^, w^+^/ Dp(1;4)1021, y^+^, sv^spa-pol^ | | |  | | |  |  |  |
| 102-1 PEV | no | n.d. | n.d. | yw; CIGAR^mCherry,102F^, w^+^/ Dp(1;4)1021, y^+^, sv^spa-pol^ | | |  | | |  |  |  |
| 108-2 | yes | yes | yes | yw; Dp(1:4)1021, y^+^ , sv^+^ / Dp(1;4)1021, y^+^, sv^spa-pol^ | | | wild-type no indel | | |  |  |  |
| 110-1 PEV | no | n.d. | n.d. | yw; CIGAR^mCherry,102F^, w^+^/ Dp(1;4)1021, y^+^, sv^spa-pol^ | | |  | | |  |  |  |
| 112-1 | yes | n.d. | n.d. | yw; CIGAR^mCherry,102F^, w^+^, sv^spa-pol^ / Dp(1;4)1021, y^+^, sv^spa-pol^ | | | wild-type no indel | | |  |  |  |
| 112-2 PEV | no | n.d. | n.d. | yw; CIGAR^mCherry,102F^, w^+^/ Dp(1;4)1021, y^+^, sv^spa-pol^ | | |  | | |  |  |  |
| 115-1 | yes | n.d. | n.d. | yw; CIGAR^mCherry,102F^, w^+^, sv^spa-pol^ / Dp(1;4)1021, y^+^, sv^spa-pol^ | | | wild-type no indel | | |  |  |  |
| 115-2 | yes | n.d. | n.d. | yw; CIGAR^mCherry,102F^, w^+^, sv^spa-pol^ / Dp(1;4)1021, y^+^, sv^spa-pol^ | | | wild-type no indel | | |  |  |  |
| 117-1 | yes | n.d. | n.d. | yw; CIGAR^mCherry,102F^, w^+^, sv^spa-pol^ / Dp(1;4)1021, y^+^, sv^spa-pol^ | | | wild-type no indel | | |  |  |  |
| 120-1 PEV | no | n.d. | n.d. | yw; CIGAR^mCherry,102F^, w^+^/ Dp(1;4)1021, y^+^, sv^spa-pol^ | | |  | |  |  |  |  |
| 121-1 PEV | no | n.d. | n.d. | yw; CIGAR^mCherry,102F^, w^+^/ Dp(1;4)1021, y^+^, sv^spa-pol^ | | |  | |  |  |  |  |
| 122-1 PEV | no | n.d. | n.d. | yw; CIGAR^mCherry,102F^, w^+^/ Dp(1;4)1021, y^+^, sv^spa-pol^ | | |  | |  |  |  |  |
| 129-1 PEV | no | n.d. | n.d. | yw; CIGAR^mCherry,102F^, w^+^/ Dp(1;4)1021, y^+^, sv^spa-pol^ | | |  | |  |  |  |  |
| 131-1 PEV | no | n.d. | n.d. | yw; CIGAR^mCherry,102F^, w^+^/ Dp(1;4)1021, y^+^, sv^spa-pol^ | | |  | |  |  |  |  |
| 132-1 PEV | no | n.d. | n.d. | yw; CIGAR^mCherry,102F^, w^+^/ Dp(1;4)1021, y^+^, sv^spa-pol^ | | |  | |  |  |  |  |
